# How to improve hospital employees’ health and well-being: a staff consultation

**DOI:** 10.1186/s12913-022-08621-y

**Published:** 2022-12-06

**Authors:** Wendy Lawrence, Jasmine Hine, Daniella Watson, Julia Smedley, Karen Walker-Bone

**Affiliations:** 1 Medical Research Council Lifecourse Epidemiology Centre, Southampton General Hospital, University of Southampton, Southampton, SO16 6YD UK; 2 NIHR, Southampton Biomedical Research Centre, University Hospital Southampton NHS Foundation Trust, Southampton, UK; 3 Faculty of Medicine, National Heart and Lung Institute, Imperial College London, London, UK; 4 School of Human Development and Health, Faculty of Medicine, Global Health Research Institute, University of Southampton, Southampton, UK; 5 Occupational Health, University Hospital Southampton NHS Foundation Trust, Southampton, UK; 6 MRC Versus Arthritis Centre for Musculoskeletal Health and Work, University of Southampton, Southampton, UK; 7 Monash Centre for Occupational and Environmental Health, Monash University, Melbourne, VIC Australia

**Keywords:** Focus groups, Health and well-being initiative, Healthcare workers, Occupational health, Public hospital, Qualitative

## Abstract

**Objective:**

Explore perspectives from healthcare workers in a large public hospital (11,000 staff) on employers supporting their health and well-being.

**Methods:**

Heads of departments/services were invited to convene focus groups, facilitated by a moderator using a semi-structured discussion guide.

**Results:**

Over 450 members of staff participated in 28 focus groups. Themes identified were: 1)unique nature of working in a large hospital, 2)hospital management agenda and relationship with staff, 3)working environment, and 4)staff health and well-being initiatives.

**Conclusions:**

Optimal uptake of health-promoting initiatives was hindered in part due to lack of staff awareness and a range of barriers. Key requirements for improving staff health were perceived to be sufficient staffing, time and space to work safely and comfortably. Engaging with staff to hear their views, build trust and identify their needs is an essential first step.

**Supplementary Information:**

The online version contains supplementary material available at 10.1186/s12913-022-08621-y.

## Introduction

In their statement, June 2022, the World Health Organisation recognised the vital role healthcare workers play in improving health service coverage and maximising the right of everybody to the highest attainable standards of health [1]. However, they highlighted that this workforce (estimated to be 65 million globally) will have a projected shortfall of 15 million by 2030. Whilst the majority of the projected shortfall is likely to be in low- and middle-income countries, they emphasised problems with education, employment, deployment, retention, and performance of healthcare workers throughout all countries, particularly when funded publicly which can cause budgetary restriction. The National Health Service (NHS) is the publicly funded healthcare system in England, and the second largest single-payer healthcare system in the world [2]. As an organisation, the NHS consists of a huge, diverse workforce of > 1 million people including clinical and non-clinical teams. Hospitals are some of the largest organisations within the NHS and are the hubs in which the biggest groups of healthcare workers are clustered. Providing all of the acute care and a large proportion of all elective surgical and outpatient care, hospitals often employ hundreds, or even several thousand, healthcare workers. Each hospital could be viewed as a community with its own culture and complexities. Working patterns are varied, with shift, part-time, flexible and off-site working, and there is a range of different contexts within the workplace itself, all of which bring their own challenges for maintaining a healthy workforce [3–5].

The health and well-being of healthcare workers has been brought into sharp focus since the onset of the COVID-19 pandemic in 2020. However, declining health and increasing stress levels amongst healthcare workers was first highlighted 10–15 years before, alongside concerns about the impact of this on staff and patient outcomes [6–10]. The health of healthcare workers was shown to have a major influence on absenteeism, presenteeism and staff well-being generally [7, 8, 10]. Unfortunately, in the NHS, staff sickness absence was 27% higher than in any other UK public sector organisation [11]. The predominant cause was poor mental and physical well-being [10, 12], with an estimated 40% of NHS employees unwell with stress every year [6, 7]. High absenteeism results in an overwhelming financial burden [13]. Presenteeism is likely to be more prominent amongst healthcare workers, as staff feel pressure to not let their colleagues down [10], feel strongly committed to their role and share concerns for patient care [14]. Presenteeism impacts the whole workforce [6, 10] and increases pressure within the service affecting quality of services and financial performance [15]. The increasing work demands within healthcare, resulting from a lack of time, financial resources, increasing patient demand and workforce shortages, can have damaging effects upon psychological, social and physical well-being [8, 16], and are key factors in stress, fatigue and burn-out [17]. Certainly, even pre-pandemic, it was demonstrated that the overall health and well-being of NHS staff was deteriorating [7], with approximately 50% of the NHS workforce overweight or obese [18–20] partially as a result of poor diet and lack of physical activity throughout the working day [20]. Putting pressure on staff to come to work when unwell is associated with poorer engagement with their jobs [21], potentially leading to decreased productivity and performance, increasing the likelihood of making medical-related errors [9, 22]. Presenteeism and poor staff health can unwittingly decrease quality of care, leading to adverse effects on patient outcomes, including impact on recovery or rehabilitation [23, 24].

In recognition of the substantial evidence base, supporting staff health became a key priority for NHS management back in 2016. Acknowledging that adopting a healthier lifestyle (related to exercise, diet, smoking, alcohol) can improve all aspects of an individual’s well-being [13, 25], has led to an increase in workplace health initiatives in recent years [13, 26–28]. In line with evidence-based guidelines provided by the National Institute for Health and Care Excellence (NICE), a number of health and well-being opportunities were offered for staff aiming to improve overall health [29]. Examples include provision of height-adjustable workstations to reduce sedentary behaviour [30], free influenza vaccinations [31] and weight loss management [32]. Lack of time due to busy, stressful working environments, and financial constraints on maintaining and supporting these services were the most common barriers to their successful uptake [33, 34]. Awareness has also been difficult to achieve [16]. Previously, such initiatives have been evaluated from a management perspective, such as take-up or satisfaction with services [13, 35]. There is limited research into employees’ perspectives of workplace health culture, health initiatives and what they really want from their employer to support their health and well-being.

In 2016, NHS England funded six pilot sites to launch the ‘NHS Healthy Workforce Project’, a programme of services and support for NHS staff to support their health and well-being. One of the pilot sites was a hospital in the South of England who developed their initiative ‘LiveWell and Inspire’, offering staff five enhanced or new services: mental health support, self-referral to physiotherapy, health checks, exercise and healthy eating programmes. Overall, in the first phase: 1050 staff attended a free enhanced healthcheck; > 1000 staff self-referred for physiotherapy; over 20 courses were held for line managers training them how to promote health and well-being; de-briefing was arranged for staff in 24 different settings after acute trauma; 18 classes were held promoting physical activity and 15 resilience courses were held. Changes to the physical environment, including improving access to healthy food at work, were made across the hospital to align with the aims of the project.

Alongside the roll-out of these initiatives, a staff consultation was undertaken through a series of well-being group discussions with staff from diverse settings to gauge their perceptions of the health issues and services being offered. The aim of this consultation was to gain a workforce perspective on how a large acute hospital can best support its staff to improve their health and well-being. Little is known about why staff utilise some services and not others, or how this varies across the workforce in a large organisation such as this. Staff views were sought about management of their own health and well-being in the workplace, experiences of well-being initiatives available in the hospital, the barriers to engaging with these, and what they want going forward to support their health and well-being.

## Methods

### Design

To coincide with the roll-out of the LiveWell and Inspire initiative, a staff consultation was commissioned. A series of well-being discussion (focus) groups were held to gain a staff perspective on how the hospital can best support the workforce to improve its health and well-being. These were led by an experienced moderator using a semi-structured discussion guide, with an observer making notes of key points. A supplementary aim was to raise awareness of staff well-being and encourage departments to focus on this within regular staff meetings.

### Ethical considerations

As part of the NHS England Healthy Workforce Project, this work was classed as service improvement, so no ethical approval was required. Established ethical guidelines were followed including those governing ethical practice for psychologists. An information sheet was emailed prior to the focus group for circulation. At the start of each focus group the moderator explained about recording/transcribing and voluntary participation, ie anyone could leave the group or remain silent, and could share as much or little as they like of their experiences of using hospital services. The second series of focus groups formed part of a Masters’ project, so ethical approval was sought from the University of Southampton’s ethics committee via an online application to ERGO II (Ethics and Research Governance Online, submission ID 47,608). No names were collected and participants were assured that any names mentioned during the discussion would be removed from transcripts and reports.

### Recruitment of participants

To maximise reach across a range of staff groups, purposive and convenience sampling was adopted with no inclusion/exclusion criteria. Email contact was made to various heads of department who advised their staff group about the consultation. Focus groups were held at a time to suit each department, often taking over part of a routine staff meeting, and in a location of that department’s choosing somewhere within the hospital.

### Materials

A semi-structured discussion guide was developed and further refined between the two rounds of discussions to include a few extra questions about changes within the hospital. The final discussion guide is included as Additional file 1: Box 1.

### Data collection and analysis

All focus group discussions were audio-recorded, transcribed and analysed thematically [36]. The transcripts were read by the research team, initial codes were identified to classify the data, and then organised into themes to answer the research question “How do NHS staff think that their employers can best support their mental and physical well-being?” Using inductive coding, the research team developed a coding framework to represent the emergent themes and sub-themes. This involved some double-coding to inform iterations to the coding framework until all were satisfied that it provided a coherent framework for answering the research question.

## Results

A total of 28 focus group discussions were conducted over the course of the project with more than 450 participants from multiple hospital departments. The focus groups were run in two rounds: the first 17 groups were conducted between July 2016 and May 2017 when the hospital had recently introduced the new initiatives; a further 11 groups were conducted between July 2018 and December 2019, when the initiatives had been in place for some time. Focus groups ran for approximately 20–50 min. Table 1 provides a breakdown of where participants were drawn from.

**Table 1:** Focus group characteristics

Date	Number of participants	NHS staff department
08/07/16	8	Radiology Administration (1)
01/08/16	15	Radiology Administration (2)
13/09/16	6	Hospital Charity
18/10/16	8	Finance
14/11/16	5	Chaplaincy
29/11/16	5	Staff Support
18/12/16	21	Medicines Management Team
13/12/16	9	Centre for Biomedical Research (CBR)
23/01/17	40–50	Occupational Therapists and Therapy Technicians
01/02/17	10	Patient Safety Team
06/02/17	31	Respiratory team including Consultants
09/02/17	19	Band 6 Cancer Care Nurses
15/02/17	8	Neuroscience Heads of Department
07/03/17	12	Band 7 Neonatal Nurses
16/03/17	9	Cost Improvement and Transformation Team
21/03/17	12	SCBR Band 7 Nurses / Sisters
10/05/17	9	Wessex Academic Health Science Network
12/07/18	22	Trauma and Orthopaedics administration / clerical staff
19/09/18	22	Joint CBR and Research & Development office
25/09/18	19	Neurology administration
18/10/18	11	Maternity Operational and Strategic Team
06/11/18	9	Neonatal team
06/12/18	19	Radiology Admin (3)
10/12/18	45	Physiotherapists
14/01/19	39	Occupational Therapists
03/06/19	4	Band 7 Nurse leaders / division leaders
10/06/19	16	Senior Nursing Team
04/12/19	9	Paediatric Oncology

Thematic analysis of the transcripts revealed four main themes: 1) unique nature of working in a large public hospital, 2) hospital management agenda and relationship with staff, 3) working environment, and 4) staff health and well-being initiatives. Each of these themes and their sub-themes are illustrated with quotes below; a random sequence generator was used to number each focus group (FG) thus anonymising these data further.

### Theme 1: Unique nature of working in a large public hospital

This theme captures the sense of hopelessness some staff experience due to the hospital’s unique structure and complexity affecting their ability to engage with initiatives designed to support their health and well-being. It has four sub-themes:

#### 1a: Culture of putting others before self

NHS staff prioritise patient health and well-being above their own:

“if we have a busy day you can forget it … we always seem to be at the bottom of the pile of our priorities … we’re always thinking about other things … and then we just forget about putting ourselves first” (FG10).

#### 1b: Shift work, break patterns and extra hours

Many felt their work patterns, limited break times and long hours precluded their involvement in health initiatives:

“…so you have like half an hour lunch and then half an hour some place in the afternoon … but you’re never guaranteed that second half an hour … [you] just get swallowed up with stuff you have to do.” (FG04)

#### 1c: Health-related appointments or activities

Some highlighted the difficulty of attending health-related appointments, eg health checks, during the working day, suggesting they must be done during their own time:

“… sometimes it’s obviously quite difficult to access those services whilst you’re at work, so therefore you’d have to come in … when you’re not at work” (FG13).

There was a particular challenge related to mental health appointments as some felt uncomfortable discussing this within the work environment:

“…if you do have any kind of mental health issue … work can impact it quite a lot … on my day off would I want to come back into work to talk to someone who works where I work to talk about work?” (FG13).

#### 1d: Extra pressure as short-staffed or poorly resourced

Many highlighted how stress and pressure were caused by the lack of resources and under-staffing within the NHS:

“… all departments are short-staffed and sometimes you can get somebody in their place, but if not, the others have to pick it up.” (FG25)

### Theme 2: Hospital management agenda and relationship with staff

This theme summarises how staff feel about their relationship with hospital management and their motives and ability to address issues raised. It has four sub-themes.

#### 2a: Privacy and confidentiality

Some staff highlighted concerns about confidentiality and privacy in their dealings with their employers, particularly if utilising any mental health initiatives:

“… when you’re speaking about your mental health … I don’t think it would be confidential … back to the manager and then everyone.” (FG13)

#### 2b: Management motives

Some were suspicious of their employer’s reasons for implementing these initiatives, feeling there was a different agenda in play:

“See I’m a cynic. Are the hospital doing it because they actually care about their staff or are they doing it because of the *C-QUIN for this year? … It does feel historically that nobody really cared and now all of a sudden they do” (FG06)

*Footnote: The Commissioning for Quality and Innovation (CQUIN) framework supports improvements in the quality of services and the creation of new, improved patterns of care.

#### 2c: Positive viewpoint

Many did appreciate the hospital management’s efforts in providing health-promoting initiatives:

“I don’t think it is the hospital’s responsibility… but the fact there is stuff in place, we can access free appointments and stuff and you know help with the diet and all that sort of stuff, is great” (FG21).

#### 2d: Recognising and addressing issues

One criticism was that the hospital management had not effectively dealt with previous feedback and that these initiatives have been implemented without accounting for all staffing groups’ needs:

“I think it would be nice to see the hospital support us to do the things that we want to do, that we can accommodate in our lives, so instead of providing things that not everyone can access, why can’t they just support us to access our own things that we want to do?” (FG06)

### Theme 3. Working environment

This theme highlights how the practicalities of the workplace environment can act as barriers for involvement with the initiatives. This has four sub-themes:

#### 3a: Hospital work environment

Many felt the working environment itself had a negative impact upon mood which reduces motivation to engage with health initiatives. One example was the lack of natural lighting:

“No natural day light, especially in the winter, you go out to the dark … come in, in the dark … has to have some sort of impact long term” (FG25)

#### 3b: Lack of space

Another barrier was the confined spaces within the hospital, and also the lack of outside green space or designated ‘staff only’ spaces in which to spend their breaks:

“…If we want to go somewhere, we don’t want to go where all the patients are sat, we want to go and sit … outside” (FG11)

Some staff suggested the workplace does not have enough facilities to support some of the well-meaning initiatives:

“…it doesn’t have changing rooms, it doesn’t have showers, and it doesn’t have a space where staff can all get together.” (FG20)

#### 3c: Lack of time

An oft-cited barrier to keeping healthy was the lack of time to read the hospital’s promotional material shared via the staff intranet or emails, or to attend the organised activities:

“I guess time out to exercise, go to the gym … actually getting the time … it’s just the hours. If you’re starting early and getting home late, it’s just quite hard to fit it in” (FG19)

#### 3d: Workplace for work only

Some preferred to manage their health and well-being outside of work, in effect distinguishing between their work and home lives:

“I want to separate work and life … I don’t want my personal stuff here, in work” (FG27)

Whereas some staff welcomed the idea of having opportunities within the workplace to focus on their health and well-being:

“I think it is also a case of making it part of your working day and that does not necessarily mean you’re working” (FG23)

### Theme 4. Staff health and well-being initiatives

There was great variability in how positive or negative staff felt about the initiatives provided by the hospital.

#### 4a: “It has been really helpful”

Some felt the new initiatives provided motivation to take care of themselves, that they made it easier to do so, and were appropriate for their needs:

“… it has given me the support and the kick up the backside that I needed” (FG21).

“There is a healthy section … it is easier to choose healthy options … the temptation is reduced…” (FG23)

“They can provide you with support, whether that be counselling, telephone support, face-to-face counselling, group support … I think that’s quite good” (FG20)

#### 4b: “It’s not quite gone far enough”

There were also some negative perceptions regarding the logistics behind some initiatives, how they come and go, and the effort needed to gain access:

“you cycle in … that’s like ten people just waiting for a shower. It was also … it was key-coded, so you had to find the right person who knew the code” (FG20)

“I done some of the classes they used to do here … the yoga was cut I think, and the Zumba classes were gone. And there used to be WeightWatchers here but that’s gone as well.” (FG25)

#### 4c: Suggestions for improvement

Some staff made suggestions of how the initiatives could be improved such as making them cheaper, and improving marketing strategies to raise awareness:

“Most people I know downstairs are doing Slimming World and would be nice to make them get that cheaper …” (FG08)

“I think more active advertising, might actually help … I don’t know half the stuff that is available … so maybe a bit more of a … launch of what we do as a hospital” (FG19)

#### 4d: What staff want

There were many ideas for what would be useful and welcomed by staff, including the organisation of group activities with a purpose, such as to raise money, or more services with easier-to-use booking systems:

“…if there were a group of staff that, were going to do something and we were gonna raise some money for something in the hospital, I think that would really motivate me to do it” (FG27).

“… in your lunch break … we could book on staffnet or something like that … a service professionally for staff who perhaps don’t have time to do things, to go” (FG08).

These themes highlight the complexity of a large organisation such as a large acute hospital attempting to meet the needs of a diverse population.

## Discussion

The aim of this study was to gain understanding of how staff in a large public hospital in the South of England feel they can best be supported by their employers to look after their health and well-being. Inductive thematic analysis elicited four themes which all related to the specific nature of working within a large hospital, and the inherent expectations, constraints and opportunities that provides. The unique nature of this setting means that staff across the board put patients’ health and well-being before their own. Unsurprisingly perhaps, people decide to work in a healthcare setting as they have a desire to support the health and well-being of others. Consequently, many were not taking allocated breaks in an attempt to keep on top of their workload so that patients did not suffer unnecessarily. The pressure many felt under on a daily basis could lead to poor self-care and health behaviours. Some appeared suspicious of management motives for taking an interest in their health; speculating that the apparent interest related more to financial targets than genuine care for them. Whilst generally appreciating the efforts the hospital management have gone to in offering a range of staff-facing health services, which many had successfully and usefully accessed, some felt that these services did not meet their real needs, creating a perceived mismatch.

Previous research into workplace initiatives found that a lack of time and services ending abruptly due to insufficient funding were the most common barriers to their successful uptake [33, 34]. Our consultation with staff also highlighted these as important factors, but the range of issues raised by staff went well beyond just time and money. The nature of the work and the workplace put enormous pressure on staff to provide optimal patient care, even when not in patient-facing roles. Additional challenges to be overcome included over-crowding, staff shortages and limited access to important health-promoting influences such as fresh air, daylight, green space, staff-only relaxation areas, healthy foods and places to exercise. Previous research has shown that awareness of health and well-being services is limited; it was reported that only half of the UK’s doctors were aware of mental health well-being services being available to them [16]. Informal observation during the focus groups of both non-verbal and verbal responses to descriptions of the range of services available, highlighted that a large number of staff were unaware of all or many of them. This is despite an extensive marketing campaign undertaken internally via the staff intranet, weekly emails, internal staff social media pages, events in the hospital entrance foyer, monthly top-down management briefings, communications via the staff partnership forum and trades unions, posters and leaflet drops, and personal email invitations to attend health checks. A variety of paper based, verbal, and electronic methods were purposely used in an attempt to widen access. In addition, frequent re-iteration of messaging and feedback mechanisms were incorporated into the communications strategy. Staff shed some light on this lack of knowledge, again largely related to a lack of time to read the correspondence. Hospital management, in contrast, reported what they felt was good uptake of the various initiatives.

In 2009, the Boorman review highlighted significant opportunities for the NHS which could be achieved with reductions in workforce sick leave, thereby creating 3.4 million additional available working days a year (equivalent to an extra 14,900 staff) at a saving of £555 million [10]. Subsequent government responses included a new framework of assessment of occupational health and safety practices, a Spearhead initiative to test workplace interventions and recently the “growing occupational health” document [37]. Our results highlight that, whilst there is a focus on improving health and well-being of healthcare workers, there can appear to be a disconnect between what might be perceived as successful at an organisational level and at a workforce level. The fact that many staff reported limited or no knowledge of the initiatives on offer is troubling, particularly in view of the sustained and genuine effort that had been made by the organisation to communicate effectively and to seek staff engagement.

It has been reported before that many evaluations of workplace interventions to improve employees’ health focus on the organisation’s perspective, ignoring employees’ perspectives of workplace health culture and health initiatives [38]. This large-scale staff consultation provided a range of novel findings which can help inform how a large healthcare organisation might improve its support for staff health and well-being. In particular, we found evidence that hospital managers need to make clear to staff their motivation for providing well-being interventions, emphasising that they value the importance of the workers and their health, rather than that they are meeting external requirements which are related to markers of hospital performance and funding. Our results suggest that staff are cynical about healthcare management and more work is needed for staff to fully trust their employers. Staff pointed to the importance to them of being able to seek help for their own health and well-being in a manner that is confidential, even if the opportunity is provided on the hospital site. Staff emphasised that they did not trust this confidentiality and consequently feared stigma (particularly in relation to seeking help for mental health problems) and that they feared vulnerability and consequences for their employment prospects [39]. Regardless of the take-up of these initiatives, it would appear that the single most important thing that hospitals can do to ensure their staff’s health and well-being is to ensure they are well-supported in their roles. The role of workplace support has been recognised since the 1980s and one commonly-used model is the demand-control-support model, widely tested in the industrial sector [40]. This model highlights that workers cope well with high levels of demand providing that they have autonomy (control) and good support (from either employers or co-workers or both). Amongst healthcare workers, it was found that high levels of work-related support attenuated feelings of exhaustion and, consequently, health complaints [41]. The job demands-resources theory [42] also emphasises the importance of social support, feedback and job variation on employee engagement, health [43] and satisfaction as well as retention [44]. Taken together therefore, our findings and those of others, confirm the importance of having hospitals properly staffed with sufficient staff and resources to undertake the work whilst allowing autonomy and facilitating staff breaks.

Our findings suggest that changing social and cultural norms for health-promoting behaviours requires a large-scale team effort across the organisation [45]. Staff from all levels of the organisation could usefully model behaviours that will lead to a healthier work culture, for example, having regular breaks, taking some exercise and eating healthily [46]. But the work environment needs to be conducive to this. This is an enormous challenge in a large, complex and resource-hungry healthcare system, but it is an essential area of focus for interventions to improve and support workers’ well-being in the future. Other areas of focus for successful implementation would be good two-way communication (consulting with staff and being seen to make changes in response to suggestions from them), and finding ways for leaders to show at every opportunity how valued staff are.

Reflecting on the findings from this staff consultation prompts the question as to what success looks like for the LiveWell and Inspire project. Is it high rates of take-up of services? Importantly, what do hospital managers consider that high rates are? Other questions that need exploring in future work, could usefully include: What would success look like to staff? What work still needs to be done? Future initiatives to improve worker health and well-being could benefit from some of the principles of implementation science perhaps including process evaluation. The point of a consultation is to learn from the views expressed by those attending, and this project has provided unique insight into these. The LiveWell and Inspire initiative gave staff across the hospital the opportunity to share their views and frustrations related to their health and well-being. From the uptake data, it was clear that many had heard about the initiatives, had used the services, or heard of others who had, and appreciated the focus on their health. However, there was a substantial proportion of participants who had not heard about, or accessed, the services. Others felt their well-being was impacted by workplace factors that would not be addressed by attending an onsite exercise class for example. The findings highlight the challenge of supporting the health and well-being of staff working in such a large, diverse organisation. It is possible that better engagement with staff may lead to increased uptake of health initiatives [47]. As staff needs change, as they have done during the COVID-19 pandemic, it is important to continue engaging with them to understand how best to support them to look after their health and stay safe. During the pandemic, considerable pressure on NHS staff has been widely reported [48–50]. It is likely they have had less time to look after their own health and well-being, making the management’s role even more important.

## Strengths and limitations

It was not possible to adopt a strategic approach to sampling hence the method adopted was somewhat ad hoc. Contact details were derived from key personnel within Occupational Health and from those attending the regular hospital Healthy Workforce meetings. All such contacts were approached and those that responded were invited to take part. Unfortunately therefore, not every member of staff in the hospital would have had the opportunity to participate. Taking direction from the manager in each department as to the constitution of the groups meant that the moderator had no control over group size or time allowed for discussion, thus these factors varied considerably. It was hard to engage with everyone in the bigger groups so it is acknowledged that the minority probably did most of the talking, so no claim is made that the quotes used are representative of the specific service/department. However, large numbers of staff from a diverse range of departments/services engaged with the study – both clinical & non-clinical. Every attempt was made by the facilitators to ensure that all participants had an opportunity to share their views. Given that many groups included both clinical and non-clinical members, it was not possible to separate out our analyses by these different types of workers. However, this study provides insight into staff perspectives on the hospital setting and workplace-based initiatives.

## Conclusions

This study has highlighted some important considerations if hospitals wish to successfully implement health and well-being initiatives. Firstly, employees are cynical about employer motivation and will need significant consultation and involvement in decisions in order for them to feel able to actively engage. Staff will need absolute reassurance that their confidential medical or health-related information will not be shared with their line managers or co-workers without consent and that it will not be factored into decisions around employability. To be successful, any initiative will need to start by taking full account of work-related factors and working conditions affecting health such as emotional and physical job demands, hours of work, staffing, resources, and feasibility of taking timetabled breaks. Finally, better ways of communicating between staff and managers will need to be found. Undoubtedly this is difficult in large and complex organisations functional 24 h a day and 7 days a week but it will be fundamental to success that employees know what is available to them and that managers are seen to listen to staff and responsive to their concerns.

## Supplementary Information

**Additional file 1.**

## Data Availability

The datasets generated and/or analysed during the current study are not publicly available due to confidentiality, as we have guaranteed anonymity to individuals, their departments and the hospital, but are available from the corresponding author on reasonable request.
