# Supplementary material for: How to improve hospital employees’ health and well-being: a staff consultation
Source: BMC Health Serv Res. 2022 Dec 6;22:1488. doi: 10.1186/s12913-022-08621-y (PMC9727936; doi:10.1186/s12913-022-08621-y)
Supplement: Supplementary file 1 — Additional file 1. [file 12913_2022_8621_MOESM1_ESM.docx]

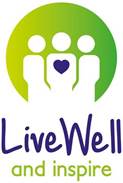


**The NHS Healthy Workforce Project**: Since 2016 UHS has provided a range of new services and sources of support for staff to encourage everyone to make healthy lifestyle changes. As well as bringing many benefits to us personally, we know that improving our own health and well-being can lead to better outcomes for our patients.

UHS would like to understand more about how staff has used these services and support, and what impact this has had on their health behaviours and overall health. To do this, we are holding a series of short discussion groups facilitated by me.

Our ambition is for staff and the Trust to work together to create initiatives that people really want, and these discussions are intended to raise awareness amongst co-workers in order to maintain engagement over the long-term. UHS feels that consulting with staff members on an ongoing basis will increase the effectiveness of the initiatives provided and the number of people wanting to access them. Everyone in the Trust has a voice, so we look forward to hearing from you.

We would like to record these discussions so that we properly capture everyone’s views. Recordings will be transcribed and no names will be left on the transcripts, so anything you say will remain anonymous. Only share what you feel comfortable sharing; if you don’t want to say anything that’s also OK – you hopefully will still find the session interesting & useful – listening to others’ experiences. We might use anonymous quotes in reports, presentations etc to illustrate what staff have been telling us.

**Discussion Guide**

**Prompt questions:**

- What do you know about the services & support offered within the Trust?
- What changes to these did you notice over the past 2 years?
- What have you (or colleagues) used?
- What were your experiences?
- What has stopped you (or others) using any of the services implemented in this scheme:
- NHS health checks
- Improved & increased physiotherapy services (inc self-referrals)
- Sports & exercise support/sessions
- Mental health support
- Eatwell sessions
- What would have made it easier?
- What else do you want?
- What will happen next?
- How will your team keep health & well-being on the agenda & thus support each other towards better health?
